# fREDUCE: Detection of degenerate regulatory elements using correlation with expression

**DOI:** 10.1186/1471-2105-8-399

**Published:** 2007-10-17

**Authors:** Randy Z Wu, Christina Chaivorapol, Jiashun Zheng, Hao Li, Shoudan Liang

**Affiliations:** 1 Department of Biochemistry and Biophysics, UCSF. 1700 4^th ^Street, San Francisco, CA 94143-2542, USA; 2 Department of Bioinformatics and Computational Biology, The University of Texas M.D. Anderson Cancer Center, 1515 Holcombe Blvd, Unit 237, Houston, TX 77030-4009, USA

## Abstract

**Background:**

The precision of transcriptional regulation is made possible by the specificity of physical interactions between transcription factors and their cognate binding sites on DNA. A major challenge is to decipher transcription factor binding sites from sequence and functional genomic data using computational means. While current methods can detect strong binding sites, they are less sensitive to degenerate motifs.

**Results:**

We present fREDUCE, a computational method specialized for the detection of weak or degenerate binding motifs from gene expression or ChIP-chip data. fREDUCE is built upon the widely applied program REDUCE, which elicits motifs by global statistical correlation of motif counts with expression data. fREDUCE introduces several algorithmic refinements that allow efficient exhaustive searches of oligonucleotides with a specified number of degenerate IUPAC symbols. On yeast ChIP-chip benchmarks, fREDUCE correctly identified motifs and their degeneracies with accuracies greater than its predecessor REDUCE as well as other known motif-finding programs. We have also used fREDUCE to make novel motif predictions for transcription factors with poorly characterized binding sites.

**Conclusion:**

We demonstrate that fREDUCE is a valuable tool for the prediction of degenerate transcription factor binding sites, especially from array datasets with weak signals that may elude other motif detection methods.

## Background

Transcriptional regulation is modulated by a complex network of interactions between regulatory proteins and their binding targets on DNA. To comprehensively understand gene regulation at a systems level, a primary goal is to decipher the "regulatory code" that consists of knowledge of all transcriptional regulators, their DNA binding profiles, and their regulatory targets [1]. Regulatory information can be inferred from the combined analysis of genomic sequence with an abundance of microarray based methods such as ChIP-chip (chromatin immunoprecipitation on microarray)[2,3] and transcription factor perturbation experiments [4,5]. However, highly reliable regulator specificies have been unattainable for many regulators probed by such genomic-scale methods [1] since weak signals from regulators are often very difficult to isolate from experimental noise.

Thus, from a computational standpoint, a major challenge is to develop techniques that can extract maximal regulator specificity information from imperfect data. A common strategy among computational tools developed for this purpose is to first obtain a small group of genes in which a given motif may be statistically over-represented, from which the motif can then be elicited using methods such as position weight matrix updating and word enumeration [6-10]. While highly effective in some cases, a potential drawback of this approach is that the process of isolating a subgroup of sequences, typically done using clustering, cutoffs, or functional categorization, can be arbitrary. The delineation of signal from background may be poor for noisy experimental data, where cutoffs can lead to significant loss of information. Other algorithms, such as dictionary- [11] or steganalysis-based [12] methods, do not rely on clustering but can benefit from subgroup selection.

A technique used by many motif-finding algorithms is to integrate expression data into the search process [12-14]. For example, the algorithm REDUCE (Regulatory Element Detection Using Correlation with Expression) avoids subgroup selection in a natural way by genome-wide fitting of motif counts to expression data [15]. REDUCE is a deterministic method that first enumerates oligonucleotides and then identifies words whose occurrence in promoter sequences correlate most strongly with expression data. This procedure is applied iteratively to produce a set of oligonucleotides that produce the best simultaneous fit to the data. REDUCE requires only a single expression dataset and makes use of the entire genomic dataset (both signal and background) to assess the significance of individual motifs. This method, which has already been widely applied [16-21], allows greater sensitivity to weak transcriptional signals and facilitates the discovery of combinatorial effects between regulators.

One weakness of REDUCE is that it can miss weak but biologically significant variants of the regulator site. Highly degenerate motifs whose individual variants fall below the detection threshold will be missed altogether. This is particularly the case for regulators in higher mammalian genomes, which can exhibit strong site to site variation in specificity. Thus, we have generalized the REDUCE approach to examine words containing degenerate IUPAC symbols representing multiple bases (i.e. S = C or G). However, a straightforward extension of REDUCE using exhaustive enumeration of degenerate motifs becomes impractical when the motif length or number of degenerate positions increase. Specifically, by including *m *IUPAC symbols in a word of length *l *the motif search space increases by a factor of $\frac{l!}{m!(l-m)!}{\left(\frac{11}{4}\right)}^{m}$ where 11 is the number of IUPAC symbols (excluding A,C,G,T). For example, the computational cost is increased by 340-fold for *l *= 10 and *m *= 2, and by 3500-fold for *m *= *3*. Therefore, we have developed fast-REDUCE (fREDUCE), a significant re-implementation of the REDUCE algorithm that allows efficient searches of the extended space of degenerate motifs. We have applied fREDUCE to detect multiple motifs for transcription factor binding sites in yeast as well as human.

## Results

### Algorithm

**Figure 1:**
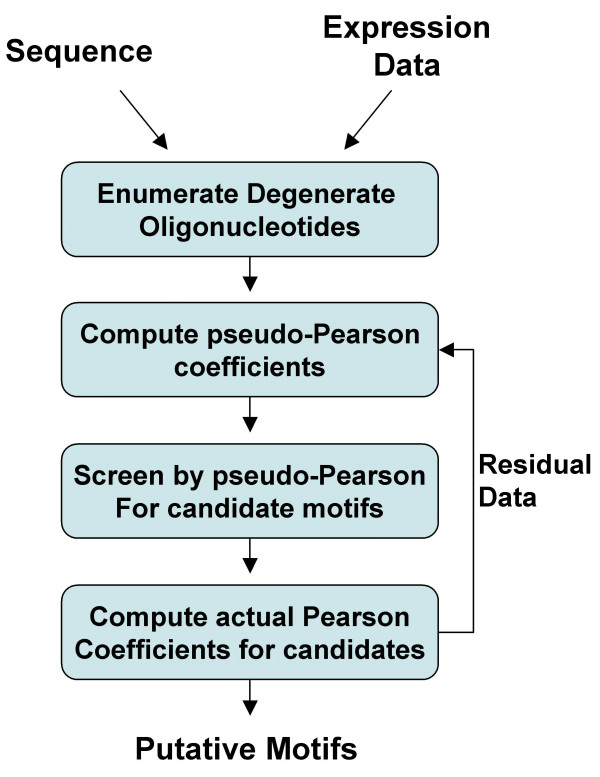
**The fREDUCE algorithm**. A set of possible IUPAC strings are generated from the input sequence. For each IUPAC string, we compute a pseudo-Pearson coefficient, which is an estimate and upper bound on the true Pearson coefficient. After the vast majority of motifs are filtered out using the pseudo-Pearson value, we then compute true Pearson coefficients for the remaining motifs and select the top motif. The residual expression value is then used to iteratively derive subsequent motifs.

The original version of REDUCE identifies motifs by exhaustively correlating all oligonucleotides up to length *l *in promoter sequences with expression data. However, the direct computation of the Pearson correlation coefficient is computationally laborious and is not well suited for analyzing large spaces of degenerate oligonucleotides. fREDUCE uses the following strategy to efficiently compute the Pearson coefficients of the most significant degenerate motifs (Figure 1): 1) A list of degenerate motifs that can be derived from the sequence data is generated. 2) For each degenerate motif, we can quickly compute a "pseudo-Pearson" coefficient, an estimate of the actual Pearson coefficient. The pseudo-Pearson coefficient is guaranteed to be an upper-bound on the actual Pearson coefficient and is used as a filter to eliminate most (typically >99.9%) of the motif list. 3) Actual Pearson coefficients are computed and the top motif is found and 4) The contribution from the top motif is subtracted from the expression data to form a residual, which is used for subsequent rounds of motif searching.

### Performance Assessment with Yeast ChIP-chip

**Figure 2:**
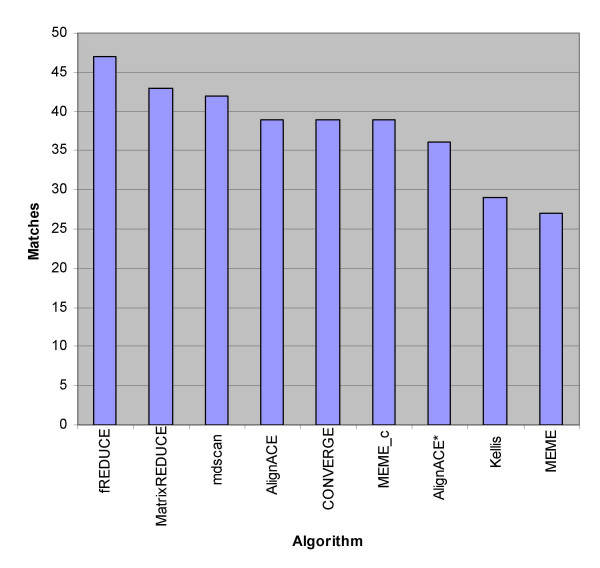
**Comparison of fREDUCE to six other algorithms on 65 yeast ChIP-chip benchmarks**. AlignACE* indicates results of running AlignACE from scratch, while the performance of other methods were compiled from the Harbison *et. al *supporting website.

To assess the performance of fREDUCE, we applied the algorithm to 352 ChIP-chip experiments from Harbison *et. al*. [1] involving 203 known and putative transcription factors in the budding yeast *S. cerevisiae*. For each ChIP-chip experiment, we correlated the normalized array data to the corresponding yeast intergenic sequences, eliciting motifs of up to length 8 and containing up to 2 IUPAC degenerate symbols. In order to verify the correctness of our predictions, we compared these results to a benchmarking set consisting of 65 high confidence motif logos assembled from the predictions of six separate motif finding algorithms [1]. For 47 of 65 benchmarks fREDUCE produced an IUPAC motif that was identical to the annotated motif, including correct degeneracies (Table 1). In comparison, we ran AlignACE [22,23] on the same 65 ChIP-chip experiments. Using the same filtering and comparison criteria, we found that AlignACE detected the annotated motif for only 36 of 65 regulators. We also compared the performance of fREDUCE with those of the other 5 motif finding algorithms used to assemble the benchmark motifs (Figure 2). Even though the benchmark motifs are likely to be biased toward the six programs from which they were originally found, fREDUCE still stood out as having the best individual performance.

**Table 1:** fREDUCE motif predictions from yeast ChIP-chip

**Factor**	**Known Site**	**Condition**	**Motif**	**p-value**	**fREDUCE match?**	**AlignACE Match?**
**ABF1**	rTCAyt....Acg	YPD	rTGATm	22.4	√	√
**ACE2**	tGCTGGT	YPD	kGCTGGy	6.2	√	
**AFT2**	GGGTGy	H2O2Lo	rGGTGy	91.5	√	√
**AZF1**	YwTTkcKkTyyckgykky	YPD	mTTTTw	14.8		
**BAS1**	TGACTC	YPD	TGACTCCG	37.2	√	√
**CAD1**	mTTAsTmAkC	YPD	GmTTAsTA	4.2	√	√
**CBF1**	tCACGTG	YPD	CACGTG	90.7	√	√
**CIN5**	TTAygTAA	YPD	TTAyrTAA	59.4	√	√
**DAL82**	GATAAGa	RAPA	GATAAG	9.4	√	
**DIG1**	TgAAAca	YPD	TGAAACA	18	√	
**FHL1**	rTGTayGGrtg	YPD	GTAyGGrT	141.2	√	√
**FKH1**	tTgTTTac	YPD	yTGTTkAC	28.8	√	
**FKH2**	aaa.GTAAACAa	YPD	GTAAACA	23.7	√	√
**GAL4**	CGG...........cCg	YPD	TTCGGAGC	4.9		√
**GAT1**	aGATAAG	RAPA	GATAAG	13.3	√	
**GCN4**	TGAsTCa	YPD	rTGAsTCA	166.7	√	√
**GLN3**	GATAAGa.a	RAPA	GATAAG	38.2	√	
**HAP1**	GGmraTA.CGs	YPD	kTTATCGG	60.3	√	√
**HAP4**	g.CcAAtcA	YPD	CCAATsAr	21.7	√	√
**HSF1**	TTCya.....TTC	H2O2Hi	TTCyrGAA	109.5	√	√
**IME1**		H2O2Hi				
**INO2**	CAcaTGc	YPD	kCACATGC	12.8	√	
**INO4**	CATGTGaaaa	YPD	CAyrTG	89.2	√	√
**LEU3**	cCGgtacCGG	YPD	CGGkACCG	10.8	√	√
**MBP1**	rACGCGt	YPD	ACGCGT	126.9	√	√
**MCM1**	tttCC.rAt..gg	Alpha	yTTCCTAA	5.7		√
**MET4**	RMmAwsTGKSgyGsc	SM	CrCGyG	14.8		
**MSN2**	mAGGGGsgg	H2O2Hi	rGGGGy	20.8	√	
**NDD1**	tt.CC.rAw..GG	YPD	CTCGAGGC	12.3		√
**NRG1**	GGaCCCT	YPD	AGGGTCs	11.3	√	√
**PDR1**	ccGCCgRAwra	YPD	CCrwACAT	11.4		
**PHD1**	sc.GC.gg	YPD	mTGCAk	21.1		√
**PHO2**	SGTGCGsygyG	Pi-				
**PHO4**	CACGTGs	Pi-	sCACGTGs	14.1	√	
**RAP1**	tGyayGGrtg	SM	GyrTGGGT	57.1	√	√
**RCS1**	ggGTGca.t	H2O2Lo	GGGTGCA	43.6	√	√
**RDS1**	kCGGCCGa	H2O2Hi	TCCGCGG	35.6	√	
**REB1**	CGGGTAA	YPD	CGGGTAAy	136.7	√	√
**RFX1**	TTgccATggCAAC	YPD	GTCGTCCG	3.2		√
**RLR1**	ATTTTCttCwTt	YPD				
**RPN4**	TTTGCCACC	H2O2Lo	TyGCCACC	109.8	√	√
**SFP1**	ayCcrtACay	SM	yCCrTACA	31.6	√	√
**SIG1**	ArGmAwCrAmAA	H2O2Hi				
**SIP4**	CGG.y.AATGGrr	SM	CTCGGCCC	58.4		
**SKN7**	G.C..GsCs	H2O2Lo	GsCyGGCC	37.7	√	
**SNT2**	yGGCGCTAyca	YPD	GrTAGCGC	96.1	√	√
**SOK2**	tGCAg..a	BUT14	GGTrCAGA	5.6		
**SPT2**	ymtGTmTytAw	YPD	TkyATA	6.2		
**SPT23**	rAAATsaA	YPD	wTkAAA	25.1		
**STB1**	rracGCsAaa	YPD	wCGCGT	4	√	
**STB4**	TCGg..CGA	YPD	CGGryCGA	7.1	√	√
**STB5**	CGGwstTAta	YPD	CGGkGwTA	24	√	
**STE12**	tgAAACa	YPD	TGAAACA	38.9	√	√
**SUM1**	gyGwCAswaaw	YPD	GyGTCAs	25.0	√	√
**SUT1**	gcsGsg..sG	YPD	wCkCCG	49.8		
**SWI4**	raCgCsAAA	YPD	CGCsAAAA	12.6	√	√
**SWI6**	tttcGCGt	YPD	TTTCsk	11.6	√	
**TEC1**	rrGAATG	YPD	rrGAATGT	22.4	√	
**THI2**	gmAAcy.twAgA	Thi-	GGAAACyS	4.5	√	
**TYE7**	tCACGTGAy	YPD	TCACGTGr	70.8	√	√
**UME6**	taGCCGCCsa	YPD	GCsGCy	154.3	√	√
**YAP1**	TTaGTmAGc	YPD	mTkACTAA	13.6	√	√
**YAP7**	mTkAsTmAk	H2O2Hi	mTTAsTAA	121.9	√	√
**YDR026c**	ttTACCCGGm	YPD	CCGGGTAA	23.2	√	√
**ZAP1**	ACCCTmAAGGTyrT	YPD	wAyATT	16.5		

fREDUCE predictions from 65 yeast ChIP-chip experiments of Harbison *et. al*. Check marks (√) indicate that fREDUCE matched the IUPAC string corresponding to the benchmark logo. The results of a similar analysis with AlignACE is given in the right column.

We also examined the performance of fREDUCE on 38 regulators for which Harbison *et. al*. detected motifs with lower confidence [see Supplementary Tables]. Noting that many of these 38 predicted motifs could contain inaccuracies, fREDUCE matched 7 of these predictions while alignACE matched 3.

### Comparision to the original REDUCE and to MatrixREDUCE

**Figure 3:**
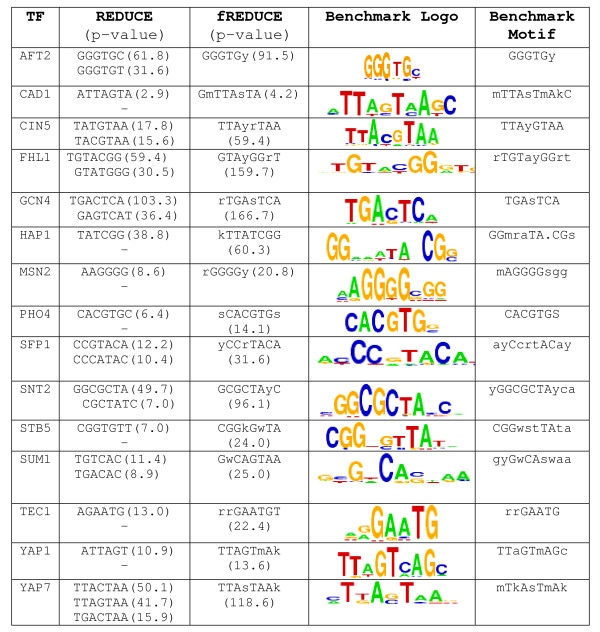
**fREDUCE predictions in comparison to non-degenerate predictions made by REDUCE**. Benchmark logos and their corresponding motifs are shown for reference. P-values are shown as -log_10 _values.

To assess the ability of fREDUCE to correctly capture motif degeneracies, we systematically compared the predictions made by fREDUCE to those made by its predecessor REDUCE on the subset of benchmark motifs containing significant degeneracy. Of 15 degenerate benchmark motifs, fREDUCE assigned IUPAC degenerate symbols identically to the benchmark in 11 cases (Figure 3). In the 4 remaining cases (HAP1, MSN2, STB5 and SUM1) fREDUCE made a prediction which is consistent with the benchmark motif while having a different degeneracy (e.g. CGGkGwTA vs. CGGwsTTA for STB5). In all of these cases, fREDUCE assigns the degenerate motif a more significant p-value than the corresponding non-degenerate motif. We note that in some cases motif degeneracies can be detected by the original REDUCE as separate motif predictions. This is especially true for regulators with strong signal (AFT2, CIN5, FHL1, GCN4, SFP1 and YAP7). However, in 5 cases degeneracies successfully predicted by fREDUCE were not detectable at all by REDUCE (CAD1, PHO4, SNT2, TEC1 and YAP1). This is typically characteristic of regulators with weaker signal.

We also compared the performance of fREDUCE to MatrixREDUCE, a recently introduced REDUCE-variant that refines motifs elicited by REDUCE into Position Specific Affinity Matrices (PSAM) [24,25]. MatrixREDUCE matched 43 of the 65 benchmarks as well as 6 of 38 motifs in the lower confidence set [see Additional file 1]. In the high confidence set, six predictions were specific to fREDUCE (HAP4, HSF1, INO4, LEU3, NFG1 and THI2) while two were specific to MatrixREDUCE (MCM1, SIP4). Specific predictions from the lower confidence set included ROX1, SWI5, UME1 for fREDUCE and PUT3, RLM1 for MatrixREDUCE. Overall, fREDUCE has a slightly stronger joint performance with 9 uniquely correct predictions from the two sets versus MatrixREDUCE's 4. In the former cases, MatrixREDUCE did not seem to begin with the correct seed, suggesting that an enumeration strategy is beneficial for some regulators. In the latter cases, fREDUCE does not find the correct motif because the long and fuzzy nature of these motifs makes them too costly for enumeration. We note that some of these differences are dependent on run parameters; with the parameters we have used MatrixREDUCE took an order of magnitude longer to run on average than fREDUCE (data not shown).

### Prediction of novel motifs from yeast ChIP-chip

Next we looked to see whether fREDUCE was capable of detecting novel motifs for transcription factors with uncharacterized specificities. Of the remaining transcription factors in the ChIP-chip study with no benchmark logo, we found 24 cases where fREDUCE made nontrivial (not repetitive poly-dA/dT sequences) motif predictions with p-values under 10^-3 ^(Table 2). In all of these cases, there has been little to no experimental information available regarding the specificity, and existing computation methods have yielded little additional insight. Nevertheless, in a few cases we found evidence in the literature which supports the novel motif predictions we have made with fREDUCE. For example, the binding site of ARO80, a regulator of the aromatic amino acid structural genes, has been characterized in two genes as being tandem repeats of the sequences TAACCG and TTGCCG [26]. From the ChIP-chip data, fREDUCE elicited the motif GATAACCG with high significance (p = 10^-41^) as well as the degenerate motif T(A/G)CCG(A/C) (p = 10^-5.6^), which is similar to both of the characterized repeat elements and reflects their degeneracies. We also considered the regulator MTH1, which negatively regulates the glucose sensing signal transduction pathway by interacting with the transcriptional repressor Rgt1p [27]. Although it is unknown whether Mth1p has intrinsic DNA sequence specificity, Rgt1p has been shown to have the specificity CGGANNA [28]. fREDUCE found the matching motif GGAGRA (p = 10^-3.57^), which is compatible with the notion that Mth1p binds to DNA in association with Rgt1p.

**Table 2:** fREDUCEpredictions for regulators with poorly characterized specificities

**Regulator**	**Predicted Site**	**P-value**	**Motif from Literature Search**
**ARG80**	TTYTCY	34.3	CYNYYAANKRMAR
**ARO80**	**TRCCGM**	**5.6**	**TWRCCG**
**ASK10**	AYTTKA	9.1	
**CST6**	TYAAWA	7.0	
**DAT1**	WTTSAA	16.7	
**ECM22**	GCRSCC	16.2	TCGTATA
**EDS1**	TWTTSA	8.4	
**FAP7**	WTRAAG	11.3	
**GAT3**	CCTSGGC	15.2	
**GCR2**	TTCAWW	5.0	CTTCC
**HAL9**	WTTRAA	14.7	
**HIR3**	WTTRAA	22.0	ACGCTAAA
**IME4**	YACACAC	17.8	
**MAL13**	CCASSG	11.6	
**MAL33**	GCRCAS	13.8	
**MET18**	WTTCAA	8.2	
**MGA1**	TTTRAY	5.9	
**MSN1**	MMCCCA	3.8	
**MTH1**	**GGAGRA**	**3.4**	CGGANNA *
**OAF1**	CGCASY	4.9	CGGNNNTNAN_9–12_CCG
**RGM1**	CSGSCC	27.1	
**RTG1**	ATYTRA	10.3	
**SIP3**	WTCAAW	7.6	
**SMK1**	WTGWAG	3.9	
**STB2**	CAAGGYC	3.1	
**STB6**	TATSAW	5.6	
**STP4**	AARMTT	24.1	
**TOS8**	RCACMC	20.7	
**UPC2**	MATSAA	4.5	
**WAR1**	TYAAGW	6.6	
**YBR239c**	WATAYT	16.8	
**YDR049W**	AWTGAW	3.5	
**YER051w**	AKYACT	3.9	
**YER130C**	CAARTW	3.1	
**YFL052w**	WTCAAK	3.6	
**YGR067C**	TTYAAW	4.6	
**YKR064W**	WGTTRA	6.3	
**YLR278C**	KTTMAA	7.2	
**YML081W**	WCAAMT	3.7	
**YNR063W**	TCAARTA	2.4	
**YPR196W**	WTCAAW	10.3	

We searched the literature for evidence supporting our motif predictions and the matching examples are highlighted. *The annotated motifs for Rgt1p.

### Motif Elicitation in Human Hepatocytes

In higher eukaryotes, motifs tend to be more degenerate and dispersed among longer intergenic regions. A common benchmark set used to evaluate the performance of computational algorithms in higher eukaryotes is the liver specific dataset [29]. Krivan et. al compiled a set of experimentally defined regulatory elements upstream of genes that were expressed exclusively in liver or in a small number of tissues including liver. From this set of genes, they found that hepatocyte-specific gene expression is mainly regulated by a small set of transcription factors (TFs), including HNF-1, HNF-3, HNF-4, and C/EBP. HNF-1, HNF-4, and C/EBP are known to be transcriptional activators based on TRANSFAC [30] annotation.

**Figure 4:**
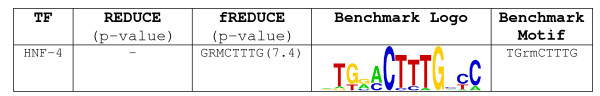
fREDUCE elicitation of the HNF-4 binding site from human hepatocyte expression data.

We ran fREDUCE on human adult hepatocyte expression data to capture binding sites of liver-specific transcription factors. fREDUCE captured both the forward and reverse complement of the HNF-4 binding site as well as two key degeneracies in the motif core as published in Krivan *et. al*. (Figure 4). HNF-4 is known to be linked to gene expression in mature liver [29], which is consistent with the expression data set used in our analysis. In contrast, REDUCE was not able to capture the known binding sites, which is most likely due to the degeneracy involved in the known consensus. These results show the potential of using fREDUCE to identify regulatory elements in higher eukaryotes, including human.

## Discussion

Despite the availability of powerful techniques such as ChIP-chip, the binding specificities of many transcription factors remain uncharacterized. This can be due to several reasons, including 1) regulators that have few genomic targets 2) regulators which interact weakly or indirectly with their targets and 3) regulators which bind to their maximal set of targets only under very specific environmental cues, which may be hard to find experimentally. fREDUCE offers increased sensitivity in these cases because it 1) uses the entire array data set for correlation and 2) searches all possible degeneracies. While fREDUCE is in some respects similar to motif regressor [14] and matrixREDUCE, a key distinction is that fREDUCE detects degenerate motifs *de novo *by exhaustive enumeration. In contrast, matrixREDUCE refines degeneracies from non-degenerate seeds and motif regressor selects among candidate matrices using correlation with expression. Thus, fREDUCE may be advantageous when motifs are difficult to detect in a non-degenerate form or are missed in the candidate set.

By comparison to 65 benchmark logos in yeast, we see that fREDUCE is comparable to or greater in detection power versus algorithms like AlignACE for strong motifs that are relatively easy to detect. Even in these cases, fREDUCE outperforms the original REDUCE algorithm by accurately predicting known degeneracies. The most advantageous use of fREDUCE, however, is for the detection of weak motifs which may lie at the border of detection. It is difficult to verify the correctness of many of the motifs elicited in these cases because of their poor characterization. Nevertheless, we have found two cases where fREDUCE was sensitive to subtle signals: ARO80, for which sites are highly degenerate, and MTH1, which may have a weak signal due an indirect interaction with DNA. We have also shown that fREDUCE is capable of capturing the HNF-4 binding site in hepatocytes, demonstrating that this algorithm is generally applicable to the detection of degenerate motifs in mammalian cells.

## Conclusion

We have presented the motif prediction algorithm fREDUCE, a refined variation of REDUCE specialized for the detection of degenerate motifs. The two primary strengths of fREDUCE are 1) it maximizes data utilization by fitting all expression data and 2) it searches motif degeneracies in a comprehensive and unbiased way. We have shown that fREDUCE is an improvement upon the existing REDUCE algorithm for degenerate binding profiles and that it can outperform existing motif finding methods on yeast ChIP-chip benchmarks. Furthermore, fREDUCE is able to detect degenerate signals in yeast and human. Thus, fREDUCE should be a valuable computation tool for the detection of subtle motifs.

## Methods

### Algorithm

The pearson correlation between expression values and counts of a possibly degenerate motif *D *is given by:

$$P(D)=\frac{\sum_{i=1}^{G}(E_{i}-\bar{E})(n_{i}^{D}-\bar{n^{D}})}{\sqrt{\sum_{i=1}^{G}\left(E_{i}^{2}-{\bar{E}}^{2}\right)}•\sqrt{\sum_{i=1}^{G}\left(n_{i}^{D}n_{i}^{D}-{\bar{n}}^{2}\right)}}$$

Where *i *is an index over genes, *E*_*i *_is the expression of gene *i*, *n*_*i*_^*D *^is the number of motif counts matching *D *in sequence *i*, $\bar{n}$ is the average of *n*_*i*_^*D *^over all genes and *G *is the total number of genes. Let *g*_*i *_be the normalized gene expression: $g_{i}=\frac{E_{i}-\bar{E}}{\sqrt{{\sum_{i=1}^{G}(E_{i}^{2}-\bar{E}}^{2})}}$, so that $\sum_{i=1}^{G}g_{i}=0$ and $\sum_{i=1}^{G}g_{i}^{2}=1$. Then the Pearson coefficient reduces to:

$$P(D)=\frac{\sum_{i=1}^{G}g_{i}n_{i}^{D}}{\sqrt{\sum_{i=1}^{G}\left(n_{i}^{D}n_{i}^{D}-{\bar{n}}^{2}\right)}}$$

Since $n_{i}^{D}=\sum_{S}n_{i}^{S}$, where the sum is over all non-degenerate nucleotide motifs *S *that match *D*, we can pre-compute and store a table of $\sum_{i=1}^{G}g_{i}n_{i}^{S}$ for all *S *and readily construct the numerator of *P(D) *for any *D*. However, the denominator is not linear in *n*_*i*_^*D *^and cannot be expressed as a sum over *S*. Nevertheless we can compute a pseudo-Pearson coefficient:

$$\tilde{P}(D)=\frac{\sum_{i=1}^{G}g_{i}n_{i}^{D}}{\sqrt{{\tilde{n}}^{2}-G{\bar{n}}^{2}}}$$

where ${\tilde{n}}^{2}=\sum_{S}\sum_{i=1}^{G}n_{i}^{S}n_{i}^{S}$ can be constructed as a sum over *S*.

Since $\sum_{i=1}^{G}n_{i}^{D}n_{i}^{D}=\sum_{i=1}^{G}\left(\sum_{S_{1}}n_{i}^{S_{1}}\right)\left(\sum_{S_{2}}n_{i}^{S_{2}}\right)=\sum_{S_{1}}\sum_{S_{2}}\sum_{i=1}^{G}n_{i}^{S_{1}}n_{i}^{S_{2}}\geq {\tilde{n}}^{2}$, we have $\left|P(D)\right|\leq \left|\tilde{P}(D)\right|$. Hence the magnitude of pseudo-Pearson coefficient is an upper bound for the magnitude of the actual Pearson coefficient, allowing rapid screening of all degenerate motifs. Actual Pearson values can then be computed for a small subset of motifs with pseudo-Pearson values above a given threshold. This scheme is effective except for motifs where ${\tilde{n}}^{2}<G{\bar{n}}^{2}$, in which case the Pearson coefficient must be computed directly. Thus, fREDUCE will give a computational advantage as long as the average motif count $\bar{n}$ is less than one.

Specifically, fREDUCE uses the following procedure:

(1) For each oligonucleotide string *S *of length *L *that appears in the sequence, we pre-compute the quantities  and ${\bar{n^{2}}}^{S}=\sum_{i=1}^{G}n_{i}^{S}n_{i}^{S}$

(2) We generate a list of all possible nucleotides containing up to *l *degeneracies matching the set of *S*.

(3) We rapidly compute corresponding quantities for all degenerate strings *D *matching *S*: , and ${\tilde{n}}^{2}=\sum_{S}\sum_{i=1}^{G}n_{i}^{S}n_{i}^{S}=\sum_{S}{\bar{n^{2}}}^{S}$ and use them to construct the pseudo-Pearson coefficient $p_{D}/\sqrt{{\tilde{n}}^{2}-G{\bar{n}}^{2}}$. We save only those motifs whose pseudo-Pearson coefficients exceed a threshold corresponding to the p-value cutoff for its motif class. For the motifs whose pseudo-Pearson coefficients cannot be calculated directly (because ${\tilde{n}}^{2}\leq G{\bar{n}}^{2}$), we compute the true Pearson.

(4) We sort the remaining motifs in decreasing order of the magnitudes of their pseudo-Pearson and compute true Pearson coefficients in this order. We stop computing when the magnitude of the pseudo-Pearson value of the current motif in the list falls below the magnitude of the true Pearson coefficient of the top motif.

(5) Finally, we compute the residual gene expression ${\tilde{g}}_{i}=g_{i}-P(D)n_{i}^{D}$, that is, the expression data after the effect of motif *D *has been taken into account. After a renormalization, the residual is used to carry out subsequent rounds of motif finding.

To estimate the statistical significance of motifs, we note that since |P(D)|<<1, its distribution is well approximated by a Normal distribution. We convert P(D) into a z-score:

$$Z(D)=P(D)\sqrt{\frac{G-2}{1-P{\left(D\right)}^{2}}}$$

This z-score is used to derive the p-value [15]:

$$pvalue=\frac{2}{\sqrt{2\pi }}\int_{Z(D)}^{\infty }e^{\frac{-t^{2}}{2}}dt$$

To correct for multiple testing, we first apply a Bonferroni correction factor of $\left(\begin{array}{c} L\\ m \end{array}\right)D^{m}4^{L-m}$ to each motif of length *L *containing *m *IUPAC symbols. This factor corresponds to the total number of motifs in the class of *L *and *m*, where *D *= 11 or 15 depending on whether 3-fold IUPAC symbols are included. We then apply a second correction factor for the total number of motif classes examined for a particular run. For example, with the settings (L = 7 and m = 1) we would examine all motifs in the classes (6,0), (6,1), (7,0) and (7,1) giving a second correction factor of 4 for each motif (we require a minimum motif length of 6). This weighted method of correction has the advantage of accounting for the fact that motif classes with larger values of *L *and *m *tend to give higher numbers of false positives.

### fREDUCE performance testing

We ran fREDUCE on the REB1_YPD ChIP-chip data from Harbison et. al. for varying *L *and *m *on an 2.40 GHz Intel Xeon processor [see Additional file 2]. In all runs, the known Reb1p binding site CGGGTAA or close variants appeared as the top motif (data not shown).

### Motif Detection from Yeast ChIP-chip

We applied fREDUCE to 354 yeast ChIP-chip experiments involving 203 known and putative transcription factors [1]. Each experiment was analyzed with fREDUCE using the corresponding set of yeast intergenic sequences, searching all motifs up to length 8 containing up to 2 two-fold IUPAC degenerate symbols. We filtered the set of motifs found for each fREDUCE run by three criteria. First, since yeast intergenic sequences have relatively low G/C content, we eliminated motifs which only contained the letters A/T/W as such motifs tend to have inflated correlation coefficients. From the remaining list of motifs, we chose the top three most significant motifs for further comparison. Accounting for the fact that we are eliciting motifs from several hundred experiments, we also discarded motifs with corrected p-values less significant than 10^-2^. If the given transcription factor was associated with ChIP-chip data under multiple environmental conditions, then filtered motifs from all conditions were combined and the top three chosen. The final motifs for each transcription factor were compared to reference motifs predicted by Harbinson *et. al*. based on a composite of several motif finding algorithms [1]. There were a total of 102 reference motifs from the authors' website [31], 65 of which were considered high confidence. Each reference motif was compared to their corresponding fREDUCE predictions using a sliding window string comparison. Predicted motifs are considered a match if there is at least one window where all IUPAC characters are consistent between both strings. Motif predictions made for transcription factors with no reference motifs were compared to literature.

### Comparison to non-degenerate REDUCE

From the 65 high confidence benchmarks, we selected cases where the annotated motif had at least one IUPAC character. In 15 of these cases, both fREDUCE and REDUCE made correct, if not correctly degenerate predictions. In 11 of these 15 cases fREDUCE made the correct IUPAC assignments. For each of these 11 cases, we considered whether the degeneracy can be assembled from non-degenerate motifs with p < 0.01 predicted by REDUCE.

### Comparison to other motif-finding algorithms

We obtained the alignACE package and ran all ChIP-chip data with the default parameters using probes with p-values below 0.001. The output alignment was converted into an IUPAC string using the method described by Cavener *et. al*. [32] and the resulting motifs were compared to reference motifs in the same way as the fREDUCE motif predictions. Details of alignACE motifs found and comparisons to alignACE motifs from Harbison *et. al*. are available in Supp. Table 1. We also obtained MatrixREDUCE [33] and ran all ChIP-chip data against the provided yeast sequence file Y5_600_Bst.fa. Default parameters were used except that we set max_motif = 10 for consistency with our fREDUCE runs. For the other five algorithms, we tallied the total number of references to each algorithm from the list of matrices on Harbison *et al*. supporting website [34].

### Motif Detection from Human Liver Tissue

158 custom made Affymetrix gene expression arrays for 79 different human tissues (2 replicates each) were obtained from Novartis in a publicly available database [35,36]. The arrays were normalized using gcrma [37,38] and the probes were annotated using Ensembl gene annotation [39] for human build 35. To study adult liver specific gene expression, we first normalized expression values for each liver tissue replicate against the average expression of all other tissues (excluding the 2 liver tissue experiments) The expression value of each gene in liver tissue experiments is represented as the following z-score:



Where *n *is the liver tissue experiment replicate number, *g *is the index over genes, *E*^ng^_liver _is the expression value of gene *g *in replicate *n*, *μ*_other _is the mean expression value of gene *g *in non-liver tissue experiments, and *σ*_other _is the standard deviation of gene *g *in non-liver tissue experiments.

Human genomic sequences (build 35) were extracted 1000 bp upstream from the transcriptional start site (TSS) if known, or from the initiation codon, based on Ensembl v35 [40]. The repeat masked promoter sequences were mapped to corresponding z-scores, which represent gene expression. This resulted in a final set of 11,710 paired z-scores and promoter sequences for input into fREDUCE. We then ran fREDUCE on the z-scores for each replicate of the liver tissue on the basis that a higher z-score translates to higher expression in liver tissues compared to the other tissues. Two different sets of parameters were run on each replicate as follows: length 8 with 0 IUPAC symbols and length 8 with 2 IUPAC symbols.

## Availability and Requirements

• **Project Name: **fREDUCE

• **Project Home Page: **

• **Operating system: **Linux

• **Programming languages: **C++

Source code and example usage are included in the release file fREDUCE-1.1.tar.gz [see Additional file 3].

## List of Abbreviations

ChIP-on-chip: chromatin immunoprecipitation on microarray; REDUCE: regulatory element detection using correlation with expression; fREDUCE: fast regulatory element detection using correlation with expression.

## Competing interests

The author(s) declares that there are no competing interests.

## Authors' contributions

RW carried out the ChIP-chip calculations and drafted the manuscript. CC carried out the hepatocyte calculations and helped with the manuscript. JZ implemented the web interface. SL & HL conceived of the algorithm. SL implemented the algorithm. HL supervised algorithm development and data analysis. All authors read and approved the final manuscript.

## Supplementary Material

Additional file 1 Supplementary Tables. Tables showing the details of motif comparisons of fREDUCE against AlighACE and MatrixREDUCE are included here. Click here for file

Additional file 2 Supplementary Figure 1. fREDUCE performance scaling as the motif length and number of degeneracies allowed is varied. Click here for file

Additional file 3 fREDUCE-1.1.tar.gz. fREDUCE source Click here for file
