# Supplementary material for: fREDUCE: Detection of degenerate regulatory elements using correlation with expression
Source: BMC Bioinformatics. 2007 Oct 17;8:399. doi: 10.1186/1471-2105-8-399 (PMC2174516; doi:10.1186/1471-2105-8-399)
Supplement: Additional file 1 — Supplementary Tables. Tables showing the details of motif comparisons of fREDUCE against AlighACE and MatrixREDUCE are included here. [file 1471-2105-8-399-S1.doc]

**Supplementary Table 1. fREDUCE performance on 38 regulators with lower confidence from Harbison *et al.***

| **Factor** | **Known Site** | **Condition** | **Motif** | **p-value** | **fREDUCE match?** | **AlignACE match?** |
| --- | --- | --- | --- | --- | --- | --- |
| ADR1 | .GGrGk | SM | TkArAG | 4.6 |  |  |
| ARR1 | TTACTAA | YPD | wTTmAA | 8.4 |  |  |
| ASH1 | yTGACT | YPD | CSCSGC | 24.2 |  |  |
| DAL80 | GATAA | RAPA | wTsAAA | 3.1 |  |  |
| DAL81 | AAAAGCCGCGGGCGGGATT | SM | CGGssC | 49.3 |  |  |
| GAL80 | CGG...........CCG | YPD | rTrATT | 5.4 |  |  |
| GCR1 | GGCTTCCwC | YPD | yATATm | 4.8 |  |  |
| GZF3 | GATAAG | RAPA | wwTGAA | 8.2 |  |  |
| HAC1 | kGmCAGCGTGTC | YPD | rACACGT | 7.1 |  |  |
| HAP2 | CCAAT | RAPA | GATTGGAT | 8.1 | √ |  |
| HAP3 | CCAAT | YPD | CGGGGGCT | 10.2 |  |  |
| HAP5 | CCAAT | YPD | CGGGGGCT | 10.6 |  |  |
| MAC1 | GAGCAAA | H2O2Hi | TTTGCTCA | 3.2 | √ |  |
| MET31 | AAACTGTGG | SM | TGsCwC | 14.9 |  |  |
| MET32 | AAACTGTGG | YPD | CmCGTGA | 4.5 |  | √ |
| MOT3 | yAGGyA | SM | rmCCTA | 3.6 | √ |  |
| MSN4 | mAGGGG. | H2O2Hi | rGGGGy | 25.8 | √ |  |
| OPI1 | TCGAAyC | YPD | TCACATGC | 5.8 |  |  |
| PDR3 | TCCGCGGA | YPD |  |  |  |  |
| PUT3 | ..CGG..........CCG | YPD | kCSCsC | 14.8 |  | √ |
| RGT1 | CGGA..A | YPD | rCGyGG | 7.9 |  |  |
| RIM101 | TGCCAAG | YPD | TCAAwm | 6.2 |  |  |
| RLM1 | CTAwwwwTAG | BUT14 | ATGAwm | 2 |  |  |
| ROX1 | ysyATTGTT | H2O2Lo | ATTGTkCT | 4.4 | √ |  |
| RPH1 | CCCCTTAAGG | H2O2Hi | CsTsCT | 30.4 |  |  |
| RTG3 | GGTCAC | RAPA | rGTCAT | 33.9 |  |  |
| SKO1 | ACGTCA | YPD | CyCCkC | 10.2 |  |  |
| SMP1 | ACTACTAwwwwTAG | YPD | TTkmAA | 22.2 |  |  |
| STP1 | rCGGC...rCGGC | YPD |  |  |  |  |
| SWI5 | kGCTGr | YPD | kGCTGG | 6.5 | √ |  |
| UGA3 | CCG....CGG | RAPA |  |  |  |  |
| UME1 | AAkGAAA.kwA | YPD | wwTGaa | 27.6 | √ | √ |
| XBP1 | CTTCGAG | H2O2Lo | CTCGAG | 33 |  |  |
| YAP3 | TTACTAA | YPD |  |  |  |  |
| YAP5 | TTACTAA | YPD | AyCCrTAC | 16.6 |  |  |
| YAP6 | TTACTAA | H2O2Lo | GGAGAAAT | 53.8 |  |  |
| YHP1 | TAATTG | YPD | TTsAAk | 6.2 |  |  |
| YOX1 | AsAATA.TGAmr | YPD | TTTkAw | 12.1 |  |  |

**Supplementary Table 2a. Details of AlignACE motif finding for yeast ChIP-chip benchmarks.** Motifs elicited from AlignACE runs using default parameters are compared with AlignACE results from Harbison *et al.* Check marks (√) indicate matches with the reference motif while asterisks (*) indicate discrepancies between our alignACE runs and those from Harbison.

| **Factor** | **Known Site** | **Harbison Motif** |  | **Found Motif** |  |
| --- | --- | --- | --- | --- | --- |
| ABF1 | rTCAyt....Acg | RTCAytwy..ACGr | √ | TCGTnnAwArTGAT | √ |
| ACE2 | tGCTGGT |  |  |  |  |
| AFT2 | GGGTGy | a..GGGGTGys | √ | GGGTGynnnkbnss | √ |
| AZF1 | YwTTkcKkTyyckgykky |  |  |  |  |
| BAS1 | TGACTC |  | * | rrArGAGTCA | √ |
| CAD1 | mTTAsTmAkC | ATTAGTmAgC | √ | ATTAGTAAGC | √ |
| CBF1 | tCACGTG | .rTCACGTG. | √ | nrTCACGTGn | √ |
| CIN5 | TTAygTAA | TTAymTAAkC | √ | kATTAyGTAA | √ |
| DAL82 | GATAAGa |  |  |  |  |
| DIG1 | TgAAAca |  |  |  |  |
| FHL1 | rTGTayGGrtg | RTGTayGGrTg | √ | ATGTAyGGrTG | √ |
| FKH1 | tTgTTTac | yy.TTGTTTAy | √ |  | * |
| FKH2 | aaa.GTAAACAa | aAa.GTAAACAA | √ | AAwkGTAAACA | √ |
| GAL4 | CGG...........cCg | CGG.s.r.wg..skCCG..s | √ | CGGnnnAswGnnsnCCG | √ |
| GAT1 | aGATAAG |  |  |  |  |
| GCN4 | TGAsTCa | rRTGAsTCA. | √ | RATGASTCAN | √ |
| GLN3 | GATAAGa.a |  |  |  |  |
| HAP1 | GGmraTA.CGs |  | √ | GGmAATAwCGs | √ |
| HAP4 | g.CcAAtcA |  | √ | CsGsCsAATnAG | √ |
| HSF1 | TTCya.....TTC | mtrGAA......rGAA | √ | TTCTAGAAnAw | √ |
| IME1 |  |  | √ |  | * |
| INO2 | CAcaTGc |  |  |  |  |
| INO4 | CATGTGaaaa | CATGTGRAaA | √ | CATGTGAAAA | √ |
| LEU3 | cCGgtacCGG | g.sCgg.ACCGG | √ | CCGGnACCGGC | √ |
| MBP1 | rACGCGt | r.rACGCGwma | √ | ArnAAACGCGT | √ |
| MCM1 | tttCC.rAt..gg | TTTCC..Aw..GG.aa | √ | TTnCCnnwTnnGGAAA | √ |
| MET4 | RMmAwsTGKSgyGsc |  |  |  |  |
| MSN2 | mAGGGGsgg |  |  |  |  |
| NDD1 | tt.CC.rAw..GG | TYyyYCyCYy | √ | TTnCCnAAwnnGGnAAA | √ |
| NRG1 | GGaCCCT | GGACCCTk...y | √ | rnnnmAGGGTCC | √ |
| PDR1 | ccGCCgRAwra | sGCCrrAAA..r | √ |  | * |
| PHD1 | sc.GC.gg |  | √ | sCnGCrsGvkns | √ |
| PHO2 | SGTGCGsygyG |  |  |  |  |
| PHO4 | CACGTGs |  |  |  |  |
| RAP1 | tGyayGGrtg | rTGYayGGrTg | √ | RTGTAYGGRTG | √ |
| RCS1 | ggGTGca.t |  | √ | GGGTGCAnTTw | √ |
| RDS1 | kCGGCCGa |  |  |  |  |
| REB1 | CGGGTAA | ksCGGGTAAy | √ | KCCGGGTAAY | √ |
| RFX1 | TTgccATggCAAC |  | * | GTTGyCATrGnAAC | √ |
| RLR1 | ATTTTCttCwTt |  |  |  |  |
| RPN4 | TTTGCCACC | TTTGCCACC. | √ | nGGTGGCAAA | √ |
| SFP1 | ayCcrtACay | cAyCcrTACA | √ | TGTAyGGrTG | √ |
| SIG1 | ArGmAwCrAmAA |  |  |  |  |
| SIP4 | CGG.y.AATGGrr |  |  |  |  |
| SKN7 | G.C..GsCs | GGsCs.SsC.s | √ |  | * |
| SNT2 | yGGCGCTAyca | yGGCGCTAyCA | √ | YGGCGCTAYCA | √ |
| SOK2 | tGCAg..a |  | √ |  | * |
| SPT2 | ymtGTmTytAw |  |  |  |  |
| SPT23 | rAAATsaA |  |  |  |  |
| STB1 | rracGCsAaa |  |  |  |  |
| STB4 | TCGg..CGA |  | * | TCGrkCCGAr | √ |
| STB5 | CGGwstTAta |  |  |  |  |
| STE12 | tgAAACa | YGaaACA..t.tGAAAC | √ | AndnTGAAACAnA | √ |
| SUM1 | gyGwCAswaaw | GyGwCAswAAw | √ | ryGwCAswAAwT | √ |
| SUT1 | gcsGsg..sG | sssCsCSsss ??? | √ |  | * |
| SWI4 | raCgCsAAA | GaArrGAAAm ??? | √ | rACGCsAAAA | √ |
| SWI6 | tttcGCGt | y.ACGCGTtT | √ |  | * |
| TEC1 | rrGAATG |  |  |  |  |
| THI2 | gmAAcy.twAgA |  |  |  |  |
| TYE7 | tCACGTGAy | tCACGTGATg | √ | CATCACGTGA | √ |
| UME6 | taGCCGCCsa | TAGCCGCcsA | √ | TCGGCGGCTA | √ |
| YAP1 | TTaGTmAGc | kmTkACTAAK | √ | ATTAGTmAkm | √ |
| YAP7 | mTkAsTmAk | GmTkAsTAAK | √ | GmTkAsTAAT | √ |
| YDR026c | ttTACCCGGm |  | * | KKCCGGGTAA | √ |
| ZAP1 | ACCCTmAAGGTyrT |  |  | ACCTTNAGGT |  |

**Supplementary Table 2b. Details of AlignACE motif finding for lower confidence yeast ChIP-chip motifs**

| **Factor** | **Predicted Site** | **AlignACE Motif** |  |
| --- | --- | --- | --- |
| ADR1 | .GGrGk | CwTCAmvGAAnwA |  |
| ARR1 | TTACTAA | TAGTAnTAGTA |  |
| ASH1 | yTGACT | TwTCCnrAwnnGGwAA |  |
| DAL80 | GATAA | GvnAynTwGCAyCAwGGAArT |  |
| DAL81 | AAAAGCCGCGGGCGGGATT | GnnTTCGGCGGC |  |
| GAL80 | CGG...........CCG | AGCwGTkmrGCG |  |
| GCR1 | GGCTTCCwC | TAGwGGAAGCy |  |
| GZF3 | GATAAG | GmkAynTwGCAyCAwGGAArT |  |
| HAC1 | kGmCAGCGTGTC | AdGmCACGTA |  |
| HAP2 | CCAAT | GGnsnGnrynyCnnynGnGnnwnGC |  |
| HAP3 | CCAAT | TGAArAATTs |  |
| HAP5 | CCAAT | GnmsnsnGCnGGGC |  |
| MAC1 | GAGCAAA | GnAAnGGGnyATTTy |  |
| MET31 | AAACTGTGG | rAwGyGTGGC |  |
| MET32 | AAACTGTGG | AAAsTGTGGC | √ |
| MOT3 | yAGGyA | CnAdGnwnTCTTGAwnr |  |
| MSN4 | mAGGGG. | GGGnGAGrrAA |  |
| OPI1 | TCGAAyC | rwwnGnTGTTGCknA |  |
| PDR3 | TCCGCGGA | AyTTGAAwAAT |  |
| PUT3 | ..CGG..........CCG | CGGGrwksGCnnnCCG | √ |
| RGT1 | CGGA..A | GnnnnmrGAAAAAnh |  |
| RIM101 | TGCCAAG | AAAAwmTGCT |  |
| RLM1 | CTAwwwwTAG | AAGArAArGG |  |
| ROX1 | ysyATTGTT | GnnGnCsnGCsGmknCG |  |
| RPH1 | CCCCTTAAGG | TGATGTGTGm |  |
| RTG3 | GGTCAC | yGCsGArnnnCGs |  |
| SKO1 | ACGTCA | ryGyAsrnnGCGG |  |
| SMP1 | ACTACTAwwwwTAG | TGTGTGkGTGT |  |
| STP1 | rCGGC...rCGGC | TTnGCsGynGAGnG |  |
| SWI5 | kGCTGr | rACGCGAAAA |  |
| UGA3 | CCG....CGG | wGCAnCAAGGAA |  |
| UME1 | AAkGAAA.kwA | AwkGnArGGwAA | √ |
| XBP1 | CTTCGAG | GwGTGnGnGwGnGknTG |  |
| YAP3 | TTACTAA | TGTGTGTGTG |  |
| YAP5 | TTACTAA | GTGTGnGTGTG |  |
| YAP6 | TTACTAA | wGCAnCAAGGAA |  |
| YHP1 | TAATTG | wrnAAAArwAA |  |
| YOX1 | AsAATA.TGAmr | TATATwwATrTAnA |  |

**Supplementary Table 3a: MatrixREDUCE results for yeast ChIP-chip benchmarks.**

| **Factor** | **Known Site** | **Condition** | **MatrixREDUCE motif** | **MatrixREDUCE match?** | **fREDUCE match?** |
| --- | --- | --- | --- | --- | --- |
| ABF1 | rTCAyt....Acg | YPD | rTCry…..ACG | √ | √ |
| ACE2 | tGCTGGT | YPD | TsCTGG..CC | √ | √ |
| AFT2 | GGGTGy | H2O2Lo | ysGGGTGCssT | √ | √ |
| AZF1 | YwTTkcKkTyyckgykky | YPD |  |  |  |
| BAS1 | TGACTC | YPD | CsTGACTCCTCT | √ | √ |
| CAD1 | mTTAsTmAkC | YPD | ATTAGTmAGC | √ | √ |
| CBF1 | tCACGTG | YPD | C.CG.G | √ | √ |
| CIN5 | TTAygTAA | YPD | TTA.gTAA | √ | √ |
| DAL82 | GATAAGa | RAPA | yCAGAyAAGGTA | √ | √ |
| DIG1 | TgAAAca | YPD | TTCTGAAACACG | √ | √ |
| FHL1 | rTGTayGGrtg | YPD | rTGy.yGGry | √ | √ |
| FKH1 | tTgTTTac | YPD | TGTTkAC | √ | √ |
| FKH2 | aaa.GTAAACAa | YPD | GTAAACA | √ | √ |
| GAL4 | CGG...........cCg | YPD |  |  |  |
| GAT1 | aGATAAG | RAPA | GATAAG | √ | √ |
| GCN4 | TGAsTCa | YPD | r.TGAsTCAy | √ | √ |
| GLN3 | GATAAGa.a | RAPA | GATAAG..w | √ | √ |
| HAP1 | GGmraTA.CGs | YPD | T.TATCGG | √ | √ |
| HAP4 | g.CcAAtcA | YPD |  |  | √ |
| HSF1 | TTCya.....TTC | H2O2Hi |  |  | √ |
| IME1 |  | H2O2Hi |  |  |  |
| INO2 | CAcaTGc | YPD | kCACrTGs | √ | √ |
| INO4 | CATGTGaaaa | YPD |  |  | √ |
| LEU3 | cCGgtacCGG | YPD |  |  | √ |
| MBP1 | rACGCGt | YPD | ACGCGT | √ | √ |
| MCM1 | tttCC.rAt..gg | Alpha | wk.CC..Aw..GG.mA | √ |  |
| MET4 | RMmAwsTGKSgyGsc | SM |  |  |  |
| MSN2 | mAGGGGsgg | H2O2Hi | mrGGGGy | √ | √ |
| NDD1 | tt.CC.rAw..GG | YPD |  |  |  |
| NRG1 | GGaCCCT | YPD |  |  | √ |
| PDR1 | ccGCCgRAwra | YPD |  |  |  |
| PHD1 | sc.GC.gg | YPD |  |  |  |
| PHO2 | SGTGCGsygyG | Pi- |  |  |  |
| PHO4 | CACGTGs | Pi- | mGCACGTks | √ | √ |
| RAP1 | tGyayGGrtg | SM | rkGy.yGGr.k | √ | √ |
| RCS1 | ggGTGca.t | H2O2Lo | GGGTGCA | √ | √ |
| RDS1 | kCGGCCGa | H2O2Hi | AnkCGGCCGArG | √ | √ |
| REB1 | CGGGTAA | YPD | CGGGTAA | √ | √ |
| RFX1 | TTgccATggCAAC | YPD |  |  |  |
| RLR1 | ATTTTCttCwTt | YPD |  |  |  |
| RPN4 | TTTGCCACC | H2O2Lo | TTyGCCACC | √ | √ |
| SFP1 | ayCcrtACay | SM | mryCCrTrCmy | √ | √ |
| SIG1 | ArGmAwCrAmAA | H2O2Hi |  |  |  |
| SIP4 | CGG.y.AATGGrr | SM | CCGGCTwATTGGrArT | √ |  |
| SKN7 | G.C..GsCs | H2O2Lo | GsC..G.CC | √ | √ |
| SNT2 | yGGCGCTAyca | YPD | rGCGCGCTAyCA | √ | √ |
| SOK2 | tGCAg..a | BUT14 |  |  |  |
| SPT2 | ymtGTmTytAw | YPD |  |  |  |
| SPT23 | rAAATsaA | YPD |  |  |  |
| STB1 | rracGCsAaa | YPD | m.ACGCGm.AA | √ | √ |
| STB4 | TCGg..CGA | YPD | TrACArTCk.kwG.AGC.C | √ | √ |
| STB5 | CGGwstTAta | YPD | CAGCGGAsCTATAmk | √ | √ |
| STE12 | tgAAACa | YPD | GTCTGAAACAnG | √ | √ |
| SUM1 | gyGwCAswaaw | YPD | GyGwCAC.A | √ | √ |
| SUT1 | gcsGsg..sG | YPD |  |  |  |
| SWI4 | raCgCsAAA | YPD | CGCGAAA | √ | √ |
| SWI6 | tttcGCGt | YPD | CGCG.y | √ | √ |
| TEC1 | rrGAATG | YPD | rrGAATGT | √ | √ |
| THI2 | gmAAcy.twAgA | Thi- |  |  | √ |
| TYE7 | tCACGTGAy | YPD | AyCACsTGACG | √ | √ |
| UME6 | taGCCGCCsa | YPD | TwrCCGCs | √ | √ |
| YAP1 | TTaGTmAGc | YPD | ATTAGTmAk | √ | √ |
| YAP7 | mTkAsTmAk | H2O2Hi | mTkAsTmA | √ | √ |
| YDR026c | ttTACCCGGm | YPD | TTACCCGGm | √ | √ |
| ZAP1 | ACCCTmAAGGTyrT | YPD |  |  |  |

**Supplementary Table 3b: MatrixREDUCE results for lower confidence motifs.**

| **Factor** | **Known Site** | **Condition** | **MatrixREDUCE motif** | **MatrixREDUCE match?** | **fREDUCE match?** |
| --- | --- | --- | --- | --- | --- |
| ADR1 | .GGrGk | SM |  |  |  |
| ARR1 | TTACTAA | YPD |  |  |  |
| ASH1 | yTGACT | YPD |  |  |  |
| DAL80 | GATAA | RAPA |  |  |  |
| DAL81 | AAAAGCCGCGGGCGGGATT | SM |  |  |  |
| GAL80 | CGG...........CCG | YPD |  |  |  |
| GCR1 | GGCTTCCwC | YPD |  |  |  |
| GZF3 | GATAAG | RAPA |  |  |  |
| HAC1 | kGmCAGCGTGTC | YPD |  |  |  |
| HAP2 | CCAAT | RAPA | sGACCAATCGGr | √ | √ |
| HAP3 | CCAAT | YPD |  |  |  |
| HAP5 | CCAAT | YPD |  |  |  |
| MAC1 | GAGCAAA | H2O2Hi | CCGAkCAAAkCTGArnG | √ | √ |
| MET31 | AAACTGTGG | SM |  |  |  |
| MET32 | AAACTGTGG | YPD |  |  |  |
| MOT3 | yAGGyA | SM | mkGGGTmAGGCAA | √ | √ |
| MSN4 | mAGGGG. | H2O2Hi | mrGGGGy | √ | √ |
| OPI1 | TCGAAyC | YPD |  |  |  |
| PDR3 | TCCGCGGA | YPD |  |  |  |
| PUT3 | ..CGG..........CCG | YPD | C.kCGs.C……wCCC | √ |  |
| RGT1 | CGGA..A | YPD |  |  |  |
| RIM101 | TGCCAAG | YPD |  |  |  |
| RLM1 | CTAwwwwTAG | BUT14 | CyAwAAATAGA | √ |  |
| ROX1 | ysyATTGTT | H2O2Lo |  |  | √ |
| RPH1 | CCCCTTAAGG | H2O2Hi |  |  |  |
| RTG3 | GGTCAC | RAPA |  |  |  |
| SKO1 | ACGTCA | YPD |  |  |  |
| SMP1 | ACTACTAwwwwTAG | YPD |  |  |  |
| STP1 | rCGGC...rCGGC | YPD |  |  |  |
| SWI5 | kGCTGr | YPD |  |  | √ |
| UGA3 | CCG....CGG | RAPA |  |  |  |
| UME1 | AAkGAAA.kwA | YPD |  |  | √ |
| XBP1 | CTTCGAG | H2O2Lo |  |  |  |
| YAP3 | TTACTAA | YPD |  |  |  |
| YAP5 | TTACTAA | YPD |  |  |  |
| YAP6 | TTACTAA | H2O2Lo |  |  |  |
| YHP1 | TAATTG | YPD |  |  |  |
| YOX1 | AsAATA.TGAmr | YPD |  |  |  |
