# Supplementary material for: fREDUCE: Detection of degenerate regulatory elements using correlation with expression
Source: BMC Bioinformatics. 2007 Oct 17;8:399. doi: 10.1186/1471-2105-8-399 (PMC2174516; doi:10.1186/1471-2105-8-399)
Supplement: Additional file 2 — Supplementary Figure 1. fREDUCE performance scaling as the motif length and number of degeneracies allowed is varied. [file 1471-2105-8-399-S2.ppt]

## Slide 1
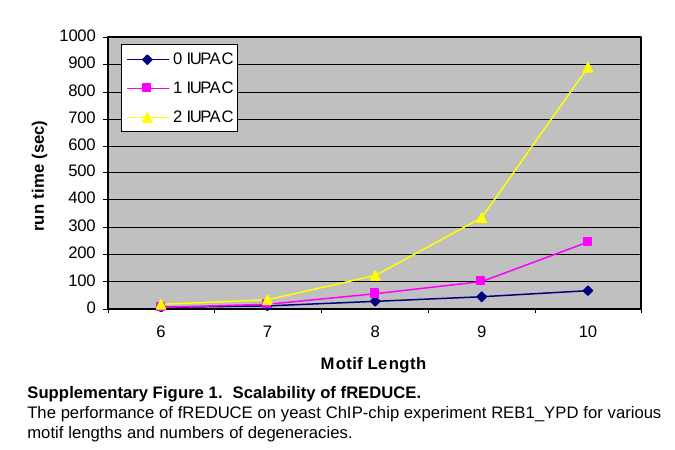

Supplementary Figure 1. Scalability of fREDUCE.
The performance of fREDUCE on yeast ChIP-chip experiment REB1_YPD for various
motif lengths and numbers of degeneracies.
